# The Effect of Sample Handling on Cross Sectional HIV Incidence Testing Results

**DOI:** 10.1371/journal.pone.0025899

**Published:** 2011-10-26

**Authors:** Oliver Laeyendecker, Amanda Latimore, Susan H. Eshleman, Jean Summerton, Amy E. Oliver, Jordyn Gamiel, Trudy Dobbs, Joanne Mei, Gary Murphy, John V. Parry, S. Michele Owen, Thomas C. Quinn

**Affiliations:** 1 Laboratory of Immunoregulation, National Institute of Allergy and Infectious Diseases, National Institutes of Health, Baltimore, Maryland, United States of America; 2 The Johns Hopkins University School of Medicine, Department of Medicine, Division of Infectious Diseases, Baltimore, Maryland, United States of America; 3 Johns Hopkins Bloomberg School of Public Health, Department of Epidemiology, Baltimore, Maryland, United States of America; 4 The Johns Hopkins University School of Medicine, Department of Pathology, Baltimore, Maryland, United States of America; 5 Centers for Disease Control and Prevention, Global AIDS Program, International Laboratory Branch, Atlanta, Georgia, United States of America; 6 Centers for Disease Control and Prevention, Newborn Screening Quality Assurance Program, Atlanta, Georgia, United States of America; 7 Microbiology Services, Health Protection Agency, London, United Kingdom; 8 Centers for Disease Control and Prevention, Division of HIV/AIDS Prevention, Atlanta, Georgia, United States of America; National University of Singapore, Singapore

## Abstract

**Objective(s):**

To determine if mishandling prior to testing would make a sample from a chronically infected subject appear recently infected when tested by cross-sectional HIV incidence assays.

**Methods:**

Serum samples from 31 subjects with chronic HIV infection were tested. Samples were subjected to different handling conditions, including incubation at 4°C, 25°C and 37°C, for 1, 3, 7 or 15 days prior to testing. Samples were also subjected to 1,3, 7 and 15 freeze-thaw cycles prior to testing. Samples were tested using the BED capture enzyme immuno assay (BED-CEIA), Vironostika-less sensitive (V-LS), and an avidity assay using the Genetic Systems HIV-1/HIV-2 plus O EIA (avidity assay).

**Results:**

Compared to the sample that was not subjected to any mishandling conditions, for the BED-CEIA, V-LS and avidity assay, there was no significant change in test results for samples incubated at 4°C or 25°C prior to testing. No impact on test results occurred after 15 freeze-thaw cycles. A decrease in assay results was observed when samples were held for 3 days or longer at 37°C prior to testing.

**Conclusions:**

Samples can be subjected up to 15 freeze-thaw cycles without affecting the results the BED-CEIA, Vironostika-LS, or avidity assays. Storing samples at 4°C or 25°C for up to fifteen days prior to testing had no impact on test results. However, storing samples at 37°C for three or more days did affect results obtained with these assays.

## Introduction

Accurate estimates of HIV-1 incidence are needed to evaluate the state of the HIV epidemic, to calibrate and validate models of epidemiology, and to plan and assess the impact of prevention interventions [1], [2]. Incidence testing provides a measure of the recent level of HIV-1 transmission in a population [2]. To obtain HIV-1 incidence estimates from cross-sectional cohorts, testing methods have been developed that take advantage of the biological differences between recently-infected and chronically-infected individuals [3], [4]. Three assays used to estimate the proportion of recently-infected individuals in a population are the BED capture enzyme immuno assay (BED-CEIA) [5], the Vironostika-less sensitive assay (V-LS) [6], and assays based on antibody avidity [7], [8], [9]. However, it is not known how serum/plasma sample handling conditions impact data generated by these incidence assays. Because incidence assays are most often performed using stored samples that may have been freeze-thawed for other investigations, it is critical to understand how prior storage and manipulation of samples might affect the data generated by cross-sectional incidence assays. We evaluated the impact of sample storage (4°C, 25°C and 37°C for up to two weeks) and freeze-thaw cycles (up to 15 cycles) on results obtained with the BED-CEIA, V-LS, and avidity assays.

## Methods

### Samples

Samples from 31 individuals with known chronic HIV infection were examined. All individuals had been HIV-infected for more than two years prior to sample collection, were not on antiretroviral drug therapy, had viral loads ≥10,000 copies/ml and had CD4 cell counts ≥200 cells/µl (range 209 to 1293). Six samples were from South African women (subtype-C), and eight samples from Ugandan women (two subtype A and six subtype D). Samples from these African study subjects were described in a previous report [10]. The remaining 17 samples were from adult patients at the Moore Clinic at Johns Hopkins University in Baltimore (subtype B). All work was conducted in accordance with the Declaration of Helsinki. All samples were delinked from personal identifiers. All participants provided written informed consent.The study was approved by the Institutional Review Boards of the Johns Hopkins University, Witwatersrand University and the Science Ethics Committee of the Uganda Virus Research Institute.

### Testing conditions

An approximate initial 1.3 ml aliquot (range 1.0 to 1.8 ml) of stored serum that had not been used for other purposes was tested. Samples were initially dispensed into as many 100 µl aliquots as the initial sample volume allowed. A control aliquot from each individual was stored at −80°C without further manipulation. The remaining aliquots from each individual were systematically subjected either to freeze-thaw cycles (3, 7, or 15 freeze-thaw cycles), or incubation at 4°C, 25°C or 37°C for specific times (1, 3, 7, or 15 days). After each manipulation, the samples were stored at −80°C until incidence testing was performed. To avoid run-to-run variation, all aliquots from a given individual were tested within the same run.

### HIV incidence testing

The BED-CEIA assay was performed according to the manufacturer's protocol (Calypte Biomedical Corporation, Lake Oswego, OR, USA) [5]. The V-LS assay was performed using a standardized optical density (SOD) cut-off of 1.0 with a 1∶20,000 diluted sera, as previously described [6]. The avidity assay was performed using the Genetic Systems HIV-1/HIV-2 Plus O EIA (Bio-Rad Laboratories, Redmond, WA) [9], [11]. To test antibody avidity samples were diluted 1∶10, incubated at 4°C for 30 minutes for the initial antibody-binding step, and then incubated with 0.1 M diethylamine for 30 minutes at 37°C for the chaotropic disassociation step. The percent avidity (Avidity Index, AI) was calculated for each sample. The AI was calculated by dividing the optical density of the DEA treated well by the optical density of the non-treated well for the same sample and multiplying by 100. All testing was performed at Johns Hopkins University.

### Analysis of HIV incidence testing data

For each study subject, we compared results from each of the three assays using the control aliquot and aliquots that were subjected to each type of freeze-thaw or storage manipulation. Paired t-tests were used to determine whether there was a significant change from the control aliquot for each type of manipulation. Statistical analyses were performed using Stata version 10.1.

## Results

A total of 428, 381 and 376 samples from the original 31 chronically infected patients were tested using BED-CEIA, V-LS, and avidity assays, respectively. Total samples available for each test differed because of the difference in the initial sample volumes available. For the BED-CEIA, the median normalized optical density (OD-n) of the untreated samples was 3.05 (interquartile range [IQR]: 2.26, 3.22). For the V-LS assay, a median SOD value for untreated samples was 4.54 (IQR: 3.67, 5.19). Samples tested using the avidity assay had a baseline median percent avidity (AI) of 100% (IQR: 78.7%, 100.0%).

Results from the BED-CEIA, V-LS, and avidity assays are shown in Table 1 and Figures S1, S2, S3, S4, S5, S6, S7, S8 and S9. The impact of freeze thaw cycles on testing is shown in Table 2 and Figures S10, S11, and S12. With the exception of conditions in which samples were stored at 37°C, there were no significant differences in results obtained using the control vs. manipulated samples. For samples stored at 37°C a decrease in testing values was observed using the BED-CEIA and V-LS assays. The mean decrease in OD-n for the BED-CEIA was 0.19 for 3 days, 0.34 for 7 days and 0.54 for 15 days. A similar trend was seen with the V-LS assay as the SOD values changed when the samples were incubated at 37°C prior to testing, with a mean decrease of 0.21 for 3 days, 0.71 for 7 days and 0.96 for 15 days. For the avidity assay, a nonsignificant decrease in AI was seen for samples incubated at 37°C prior to testing, with a mean decrease 1.41 for 3 days, 7.71 for 7 days and 1.99 for 15 days.

**Table 1:** Mean Change in Assay Results by Handling Condition.

Days incubated prior to testing	1		3		7		15	
	N	Mean Change (SD)	N	Mean Change (SD)	N	Mean Change (SD)	N	Mean Change (SD)
**BED assay (OD-n)**								
Storage at 4°C	30	−0.05 (±0.59)	31	−0.01 (±0.23)	31	0.02 (±0.26)	27	−0.01 (±0.31)
Storage at 25°C	30	0.02 (±0.25)	32	−0.03 (±0.22)	31	−0.02 (±0.12)	27	−0.05 (±0.23)
Storage at 37°C	31	−0.02 (±0.21)	28	−0.19 (±0.29)†	19	−0.34 (±0.27)†	6	−0.54 (±0.20)‡
**Vironostika-LS assay (SOD)**								
Storage at 4°C	31	0.08 (±0.33)	31	0.01 (±0.45)	31	0.05(±0.54)	8	0.11 (±0.58)
Storage at 25°C	30	0.13 (±0.41)	31	0.06 (±0.40)	30	0.07(±0.50)	8	−0.16 (±0.36)
Storage at 37°C	30	0.02(±0.45)	28	−0.21 (±0.50)‡	19	−0.71 (±1.33)‡	3	−0.96 (±0.33)‡
**Avidity assay** (%)								
Storage at 4°C	31	1.71 (±5.67)	31	0.36 (±8.17)	30	−1.75 (±18.9)	8	−3.41 (±10.0)
Storage at 25°C	31	−0.29 (±7.20)	31	0.89 (±4.71)	30	−3.38 (±19.1)	8	−2.54 (±8.32)
Storage at 37°C	31	−0.34 (±4.98)	28	−1.41 (±4.90)	14	−7.71 (±18.0)	3	−1.99 (±1.04)+
SD: Standard Deviation. Paired t-test: †p<.05, ‡p<.01, +p = .08								

SD: standard deviation; OD-n: normalized optical density; SOD standardized optical density;

† P value<0.05.

‡ P value<0.01.

+ P value = 0.08.

**Table 2:** Mean Change in Assay Results by Number of Freeze Thaw Cycles Prior to Testing.

Number of freeze thaw cycles prior to testing		3		7		15
	N	Mean Change (SD)	N	Mean Change (SD)	N	Mean Change (SD)
**BED assay (OD-n)**	31	−0.04 (±0.23)	30	−0.01 (±0.23)	12	−0.01 (±0.23)
**Vironostika-LS assay (SOD)**	31	0.02 (±0.41)	31	−0.01 (±0.42)	8	−0.05 (±0.50)
**Avidity assay (%)**	31	1.39 (±7.48)	30	1.86 (±5.84)	8	−4.02 (±12.7)
SD: Standard Deviation.						

Mean Change in Assay Results by Number of Freeze Thaw Cycles Prior to Testing.

SD: standard deviation; OD-n: normalized optical density; SOD standardized optical density.

## Discussion

Our analysis demonstrates that results generated using the BED-CEIA, V-LS, and avidity assays are not significantly affected by most sample-handing conditions. Freezing and thawing samples up to 15 times had no impact on the values generated by any of the three assays. Additionally, storing samples at 4°C or 25°C prior to testing had no statistically significant effect on the test results in the bivariate analyses. We did demonstrate a minor decrease in the values obtained using the BED-CEIA, V-LS, and avidity assays when samples were stored for an extended period at 37°C. Because relatively few aliquots were tested in the evaluation of the 15-day incubation and 15 freeze-thaw cycle conditions, the moderate changes observed in assay test results under those conditions were not conclusive. A larger sample size might provide stronger statistical support. These results are similar to those reported previously for the stability of samples used for testing with HIV-1 antibody assays for the diagnosis of HIV-1 infection [12]. In that study, repeated freeze-thaws and incubation at −20°C and 4°C did not alter the capacity of ELISA and Western blot assays to detect antibodies to HIV. A small decline in antibody reactivity was noted when samples were incubated at 25°C or 37°C for more than 21 days; however, that change did not alter the interpretation of the test results. [12]. Our study extends the previous study by evaluating the effect of freeze-thaws and incubation at different temperatures on the performance of assays that measure different characteristics of the anti-HIV antibody response: the proportion of antibody that is HIV-specific, antibody titer, and antibody avidity. In summary, these data indicate that stored samples previously frozen and thawed multiple times can be used for antibody-based incidence testing. Unless samples were stored at excessive temperatures for an extended period, similar results were obtained using samples subjected to a variety of freeze-thaw and incubation conditions.

## Supporting Information

Figure S1 **Effect of Days at 4°C on BED-CEIA Assay Results.** Samples from subtype C infected individuals from South Africa are marked in green. Samples from subtype A infected individuals from Uganda are marked in orange. Samples from subtype D infected individuals from Uganda are marked in red. Samples from subtype B infected individuals are marked in blue. The x-axis denotes the number of days the sample was incubated at 4°C prior to testing. The y-axis is the assay results in normalized optical density units.
(PDF)

Figure S2 **Effect of Days at 25°C on BED-CEIA Assay Results.** Samples from subtype C infected individuals from South Africa are marked in green. Samples from subtype A infected individuals from Uganda are marked in orange. Samples from subtype D infected individuals from Uganda are marked in red. Samples from subtype B infected individuals are marked in blue. The x-axis denotes the number of days the sample was incubated at 25°C prior to testing. The y-axis is the assay results in normalized optical density units.
(PDF)

Figure S3 **Effect of Days at 37°C on BED-CEIA Assay Results.** Samples from subtype C infected individuals from South Africa are marked in green. Samples from subtype A infected individuals from Uganda are marked in orange. Samples from subtype D infected individuals from Uganda are marked in red. Samples from subtype B infected individuals are marked in blue. The x-axis denotes the number of days the sample was incubated at 37°C prior to testing. The y-axis is the assay results in normalized optical density units.
(PDF)

Figure S4 **Effect of Days at 4°C on Vironostika-LS Assay Results.** Samples from subtype C infected individuals from South Africa are marked in green. Samples from subtype A infected individuals from Uganda are marked in orange. Samples from subtype D infected individuals from Uganda are marked in red. Samples from subtype B infected individuals are marked in blue. The x-axis denotes the number of days the sample was incubated at 4°C prior to testing. The y-axis is the assay results in standardized optical density units.
(PDF)

Figure S5 **Effect of Time at 25°C on Vironostika-LS Assay Results.** Samples from subtype C infected individuals from South Africa are marked in green. Samples from subtype A infected individuals from Uganda are marked in orange. Samples from subtype D infected individuals from Uganda are marked in red. Samples from subtype B infected individuals are marked in blue. The x-axis denotes the number of days the sample was incubated at 25°C prior to testing. The y-axis is the assay results in standardized optical density units.
(PDF)

Figure S6 **Effect of Days at 37°C on Vironostika -LS Assay.** Samples from subtype C infected individuals from South Africa are marked in green. Samples from subtype A infected individuals from Uganda are marked in orange. Samples from subtype D infected individuals from Uganda are marked in red. Samples from subtype B infected individuals are marked in blue. The x-axis denotes the number of days the sample was incubated at 37°C prior to testing. The y-axis is the assay results in standardized optical density units.
(PDF)

Figure S7 **Effect of Days at 4°C on Avidity Assay Results.** Samples from subtype C infected individuals from South Africa are marked in green. Samples from subtype A infected individuals from Uganda are marked in orange. Samples from subtype D infected individuals from Uganda are marked in red. Samples from subtype B infected individuals are marked in blue. The x-axis denotes the number of days the sample was incubated at 4°C prior to testing. The y-axis is the assay results as an avidity index.
(PDF)

Figure S8 **Effect of Days at 25°C on Avidity Assay Results.** Samples from subtype C infected individuals from South Africa are marked in green. Samples from subtype A infected individuals from Uganda are marked in orange. Samples from subtype D infected individuals from Uganda are marked in red. Samples from subtype B infected individuals are marked in blue. The x-axis denotes the number of days the sample was incubated at 25°C prior to testing. The y-axis is the assay results as an avidity index.
(PDF)

Figure S9 **Effect of Days at 37°C on Avidity Assay Results.** Samples from subtype C infected individuals from South Africa are marked in green. Samples from subtype A infected individuals from Uganda are marked in orange. Samples from subtype D infected individuals from Uganda are marked in red. Samples from subtype B infected individuals are marked in blue. The x-axis denotes the number of days the sample was incubated at 37°C prior to testing. The y-axis is the assay results as an avidity index.
(PDF)

Figure S10 **Freeze Thaw Cycles on BED-CEIA Assay Results.** Samples from subtype C infected individuals from South Africa are marked in green. Samples from subtype A infected individuals from Uganda are marked in orange. Samples from subtype D infected individuals from Uganda are marked in red. Samples from subtype B infected individuals are marked in blue. The x-axis denotes the number of days the sample was freeze thawed prior to testing. The y-axis is the assay results in normalized optical density units.
(PDF)

Figure S11 **Freeze Thaw Cycles on Vironostika-LS Assay Results.** Samples from subtype C infected individuals from South Africa are marked in green. Samples from subtype A infected individuals from Uganda are marked in orange. Samples from subtype D infected individuals from Uganda are marked in red. Samples from subtype B infected individuals are marked in blue. The x-axis denotes the number times the sample was freeze thawed prior to testing. The y-axis is the assay results in standardized optical density units.
(PDF)

Figure S12 **Effect of Freeze Thaw Cycles on Avidity Assay Results.** Samples from subtype C infected individuals from South Africa are marked in green. Samples from subtype A infected individuals from Uganda are marked in orange. Samples from subtype D infected individuals from Uganda are marked in red. Samples from subtype B infected individuals are marked in blue. The x-axis denotes the number of days the sample was freeze thawed prior to testing prior to testing. The y-axis is the assay results as an avidity index.
(PDF)
