# Supplementary figures and images for: The Effect of Sample Handling on Cross Sectional HIV Incidence Testing Results
Source: PLoS One. 2011 Oct 26;6(10):e25899. doi: 10.1371/journal.pone.0025899 (PMC3202521; doi:10.1371/journal.pone.0025899)

Supplementary Figure S1.

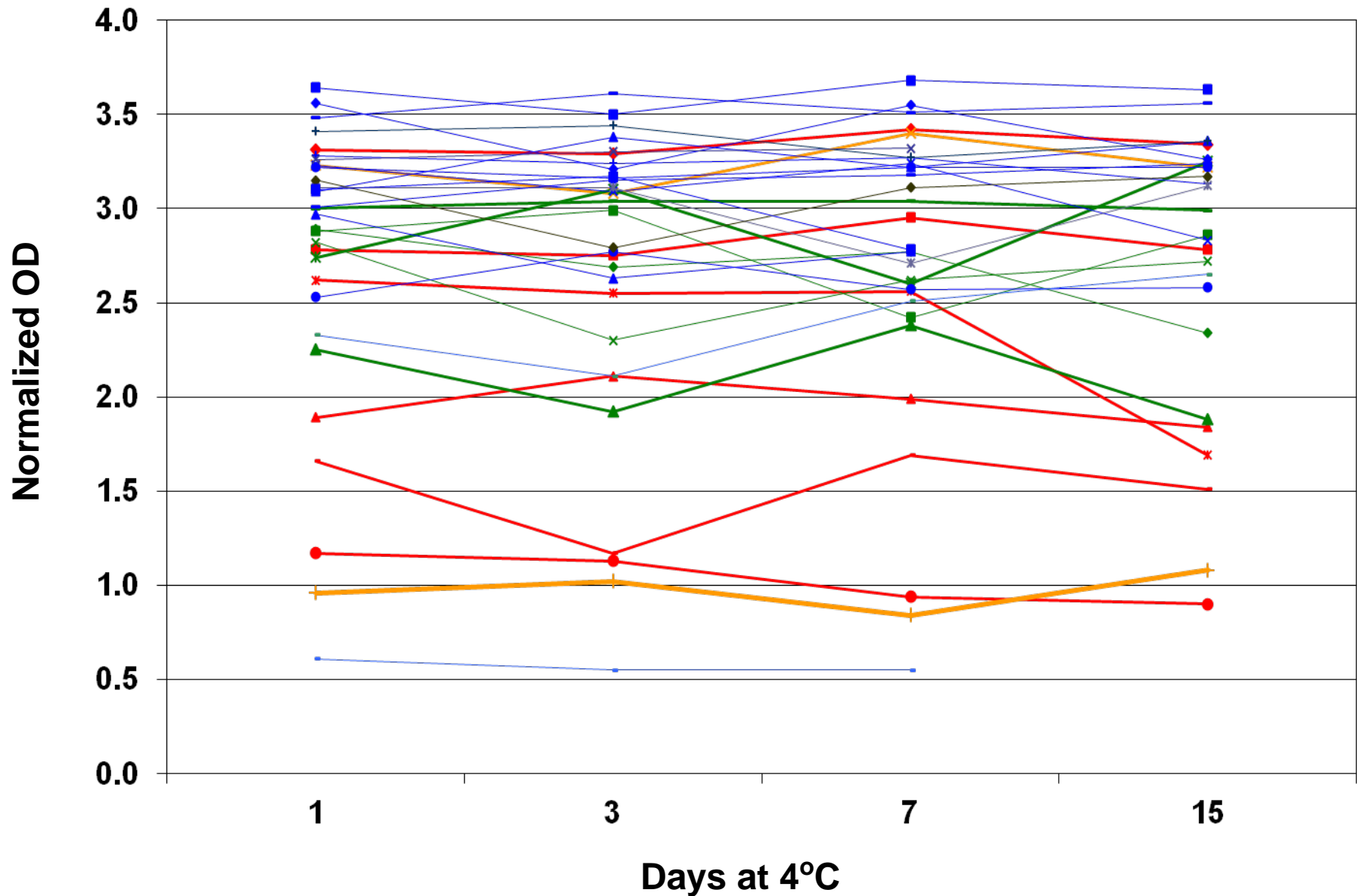

Supplement: Figure S1 — Effect of Days at 4°C on BED-CEIA Assay Results. Samples from subtype C infected individuals from South Africa are marked in green. Samples from subtype A infected individuals from Uganda are marked in orange. Samples from subtype D infected individuals from Uganda are marked in red. Samples from subtype B infected individuals are marked in blue. The x-axis denotes the number of days the sample was incubated at 4°C prior to testing. The y-axis is the assay results in normalized optical density units. (PDF) [file pone.0025899.s001.pdf]

## Supplementary Figure S2.

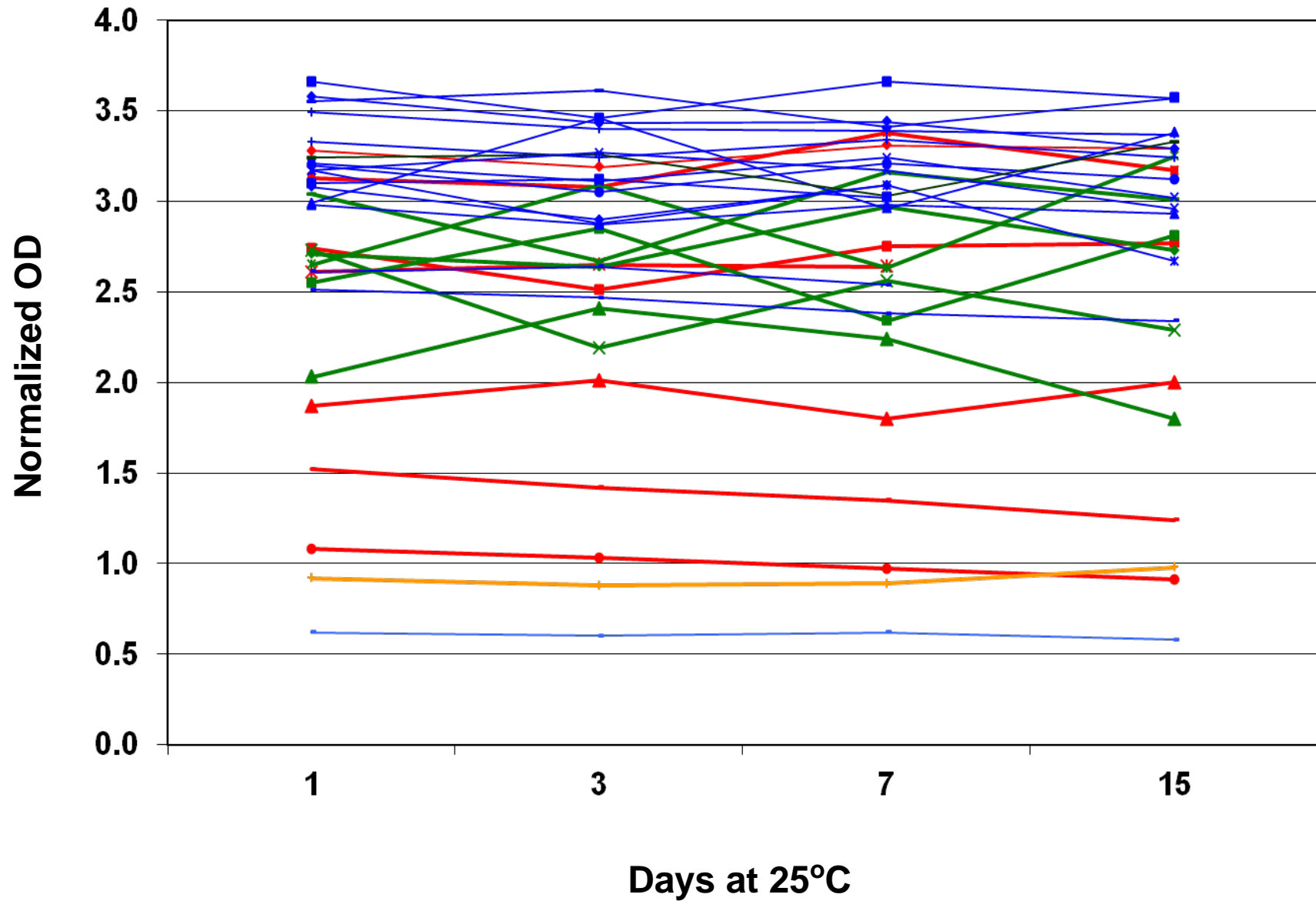

Supplement: Figure S2 — Effect of Days at 25°C on BED-CEIA Assay Results. Samples from subtype C infected individuals from South Africa are marked in green. Samples from subtype A infected individuals from Uganda are marked in orange. Samples from subtype D infected individuals from Uganda are marked in red. Samples from subtype B infected individuals are marked in blue. The x-axis denotes the number of days the sample was incubated at 25°C prior to testing. The y-axis is the assay results in normalized optical density units. (PDF) [file pone.0025899.s002.pdf]

**Supplementary Figure S3.**

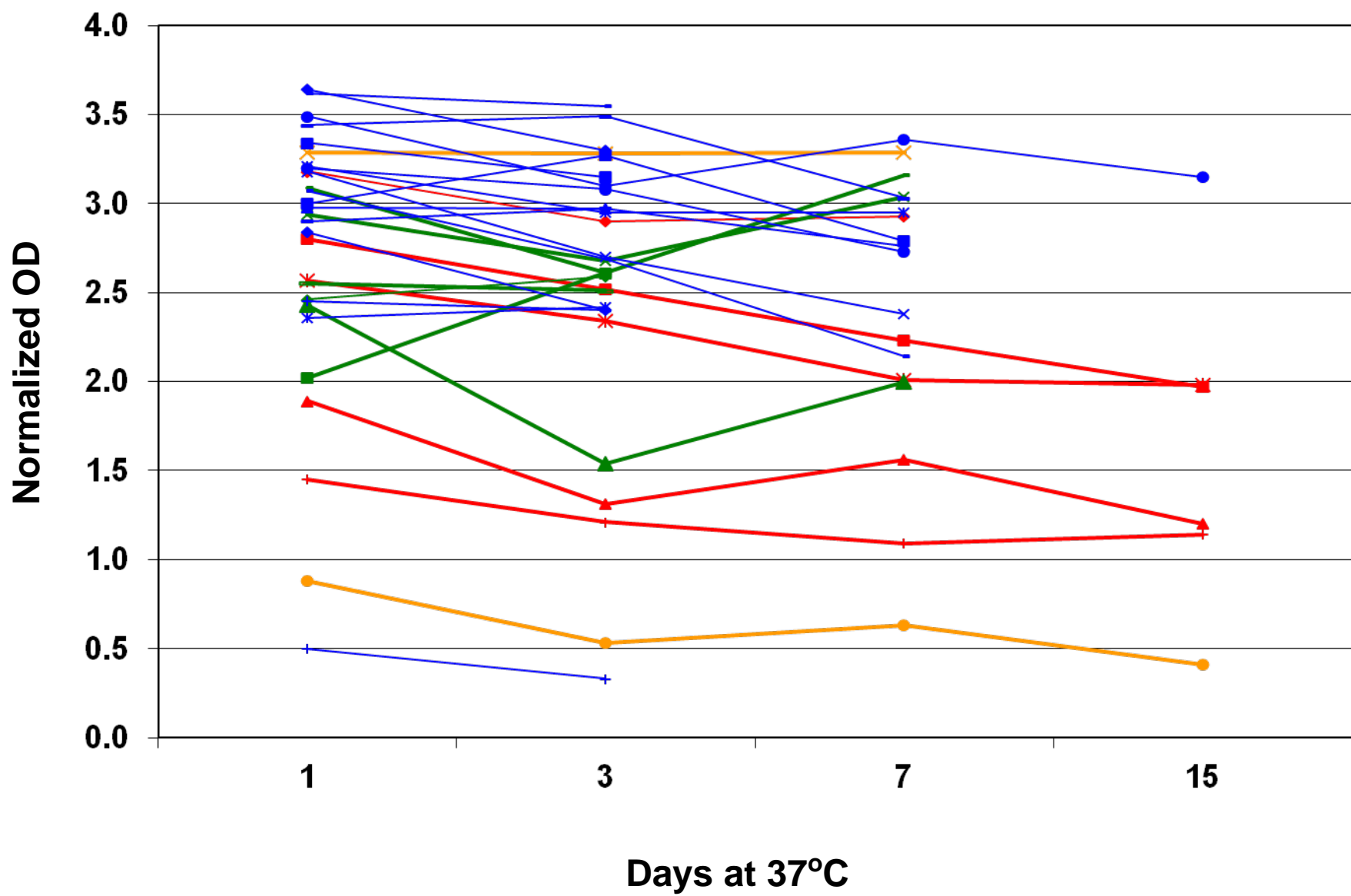

Supplement: Figure S3 — Effect of Days at 37°C on BED-CEIA Assay Results. Samples from subtype C infected individuals from South Africa are marked in green. Samples from subtype A infected individuals from Uganda are marked in orange. Samples from subtype D infected individuals from Uganda are marked in red. Samples from subtype B infected individuals are marked in blue. The x-axis denotes the number of days the sample was incubated at 37°C prior to testing. The y-axis is the assay results in normalized optical density units. (PDF) [file pone.0025899.s003.pdf]

Supplementary Figure S4.

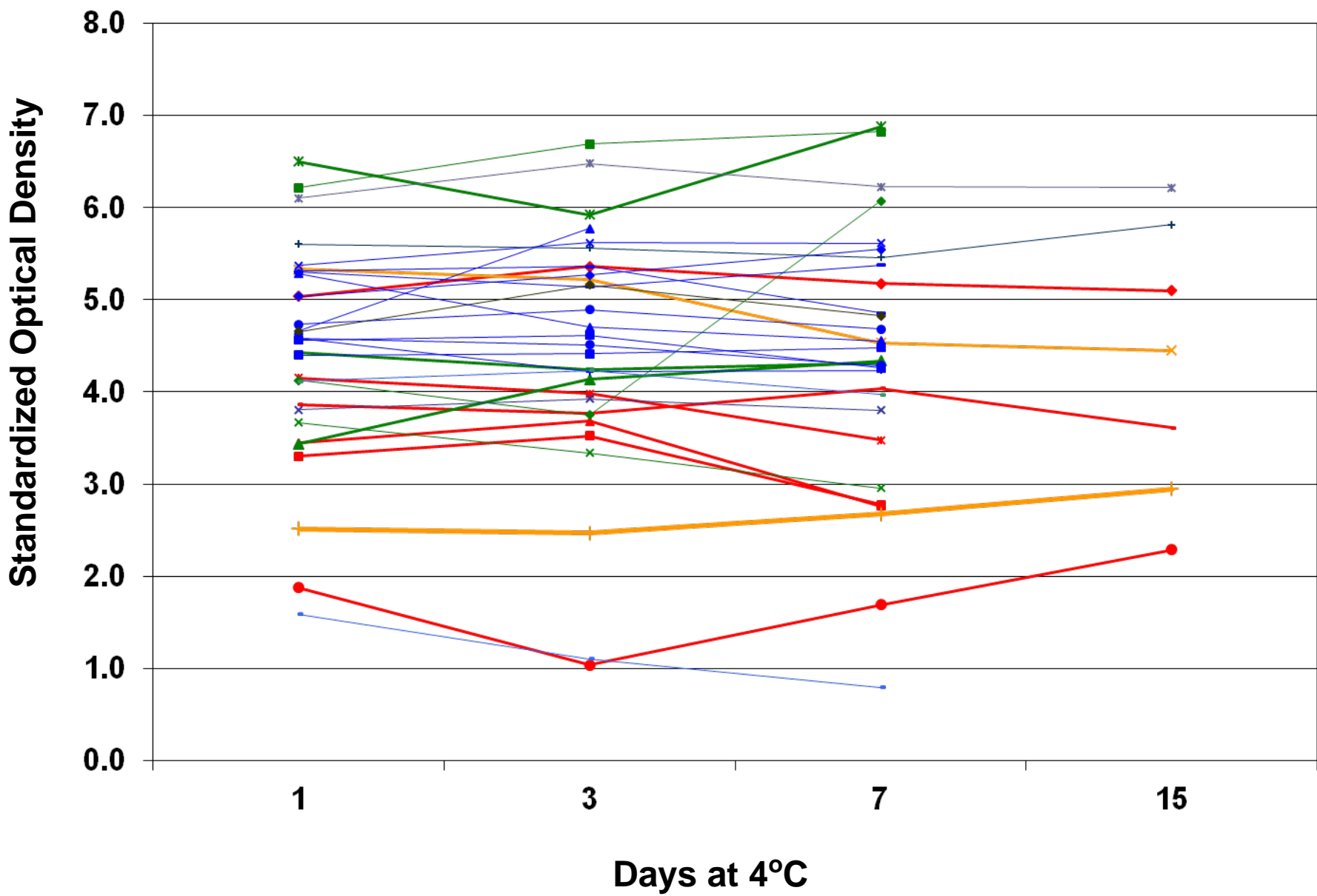

Supplement: Figure S4 — Effect of Days at 4°C on Vironostika-LS Assay Results. Samples from subtype C infected individuals from South Africa are marked in green. Samples from subtype A infected individuals from Uganda are marked in orange. Samples from subtype D infected individuals from Uganda are marked in red. Samples from subtype B infected individuals are marked in blue. The x-axis denotes the number of days the sample was incubated at 4°C prior to testing. The y-axis is the assay results in standardized optical density units. (PDF) [file pone.0025899.s004.pdf]

## Supplementary Figure S5.

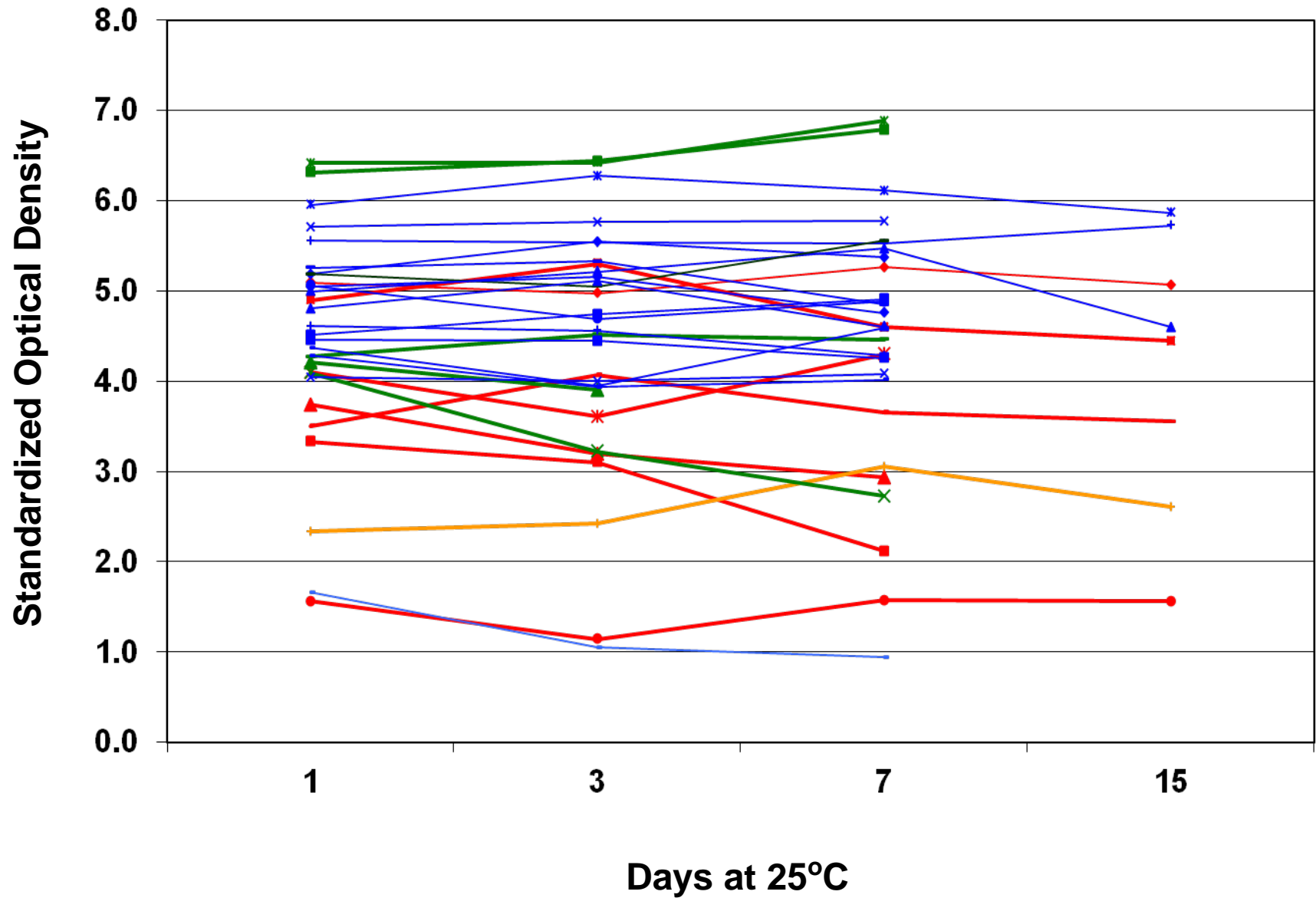

Supplement: Figure S5 — Effect of Time at 25°C on Vironostika-LS Assay Results. Samples from subtype C infected individuals from South Africa are marked in green. Samples from subtype A infected individuals from Uganda are marked in orange. Samples from subtype D infected individuals from Uganda are marked in red. Samples from subtype B infected individuals are marked in blue. The x-axis denotes the number of days the sample was incubated at 25°C prior to testing. The y-axis is the assay results in standardized optical density units. (PDF) [file pone.0025899.s005.pdf]

## Supplementary Figure S6.

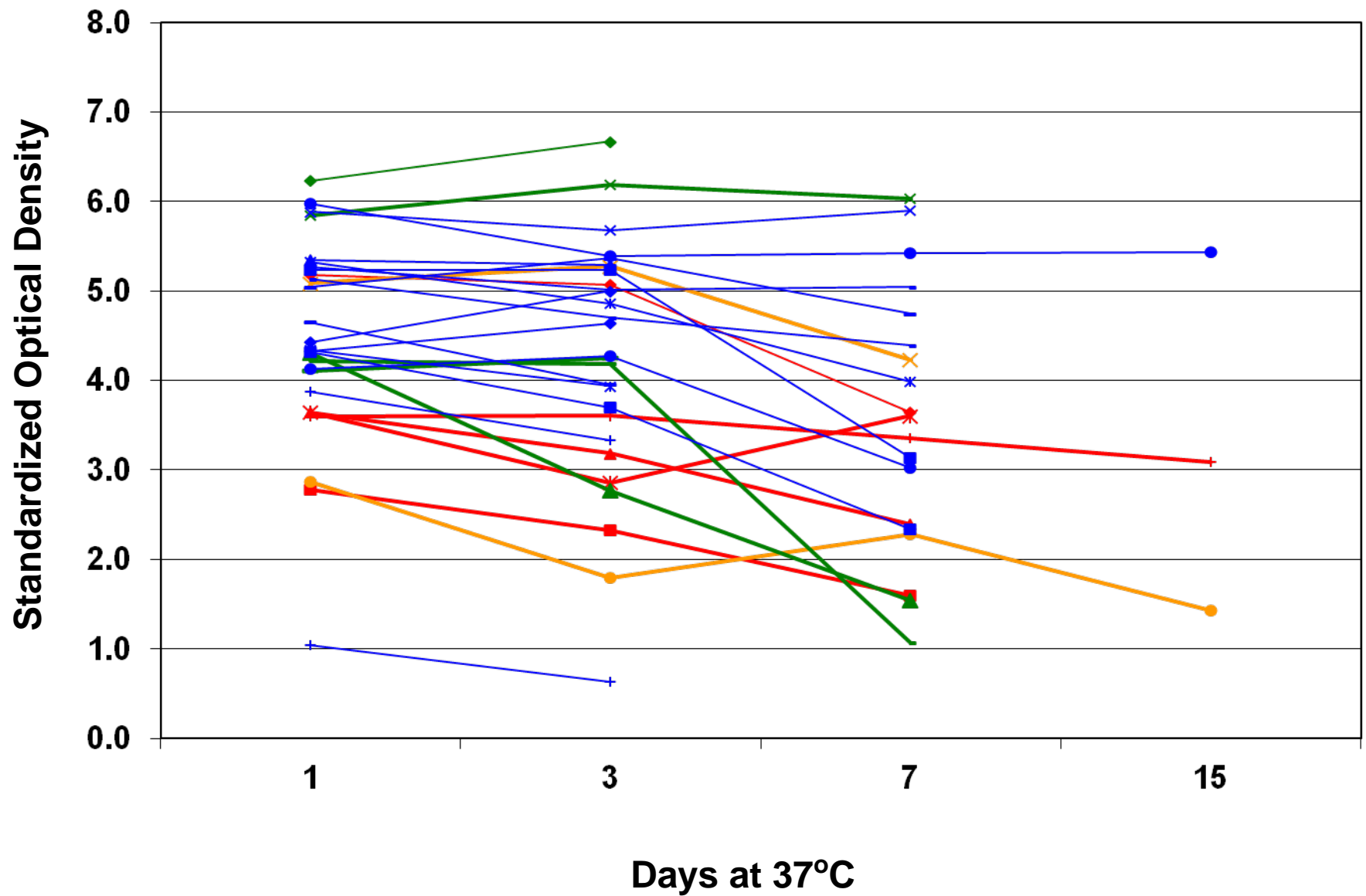

Supplement: Figure S6 — Effect of Days at 37°C on Vironostika -LS Assay. Samples from subtype C infected individuals from South Africa are marked in green. Samples from subtype A infected individuals from Uganda are marked in orange. Samples from subtype D infected individuals from Uganda are marked in red. Samples from subtype B infected individuals are marked in blue. The x-axis denotes the number of days the sample was incubated at 37°C prior to testing. The y-axis is the assay results in standardized optical density units. (PDF) [file pone.0025899.s006.pdf]

**Supplementary Figure S7.**

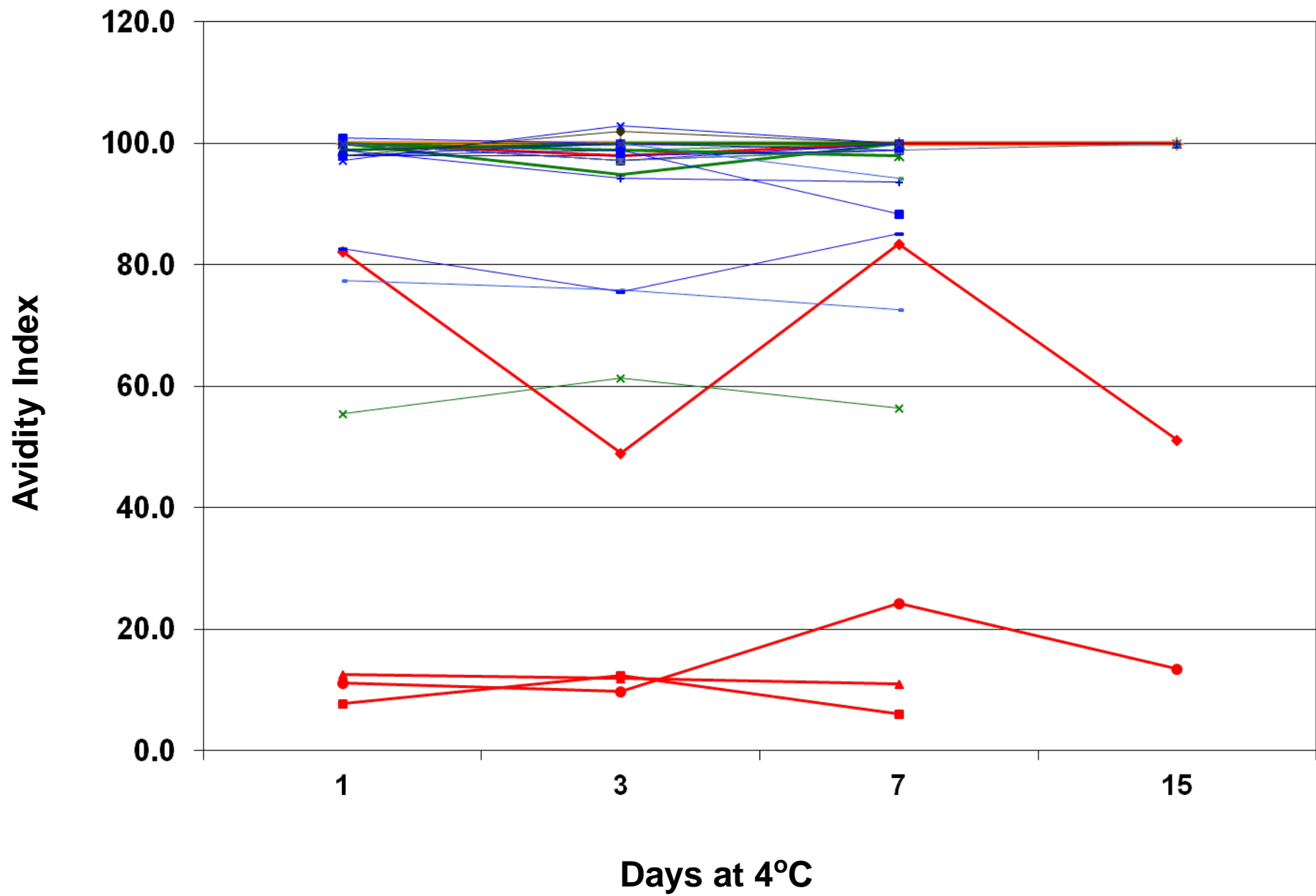

Supplement: Figure S7 — Effect of Days at 4°C on Avidity Assay Results. Samples from subtype C infected individuals from South Africa are marked in green. Samples from subtype A infected individuals from Uganda are marked in orange. Samples from subtype D infected individuals from Uganda are marked in red. Samples from subtype B infected individuals are marked in blue. The x-axis denotes the number of days the sample was incubated at 4°C prior to testing. The y-axis is the assay results as an avidity index. (PDF) [file pone.0025899.s007.pdf]

**Supplementary Figure S8.**

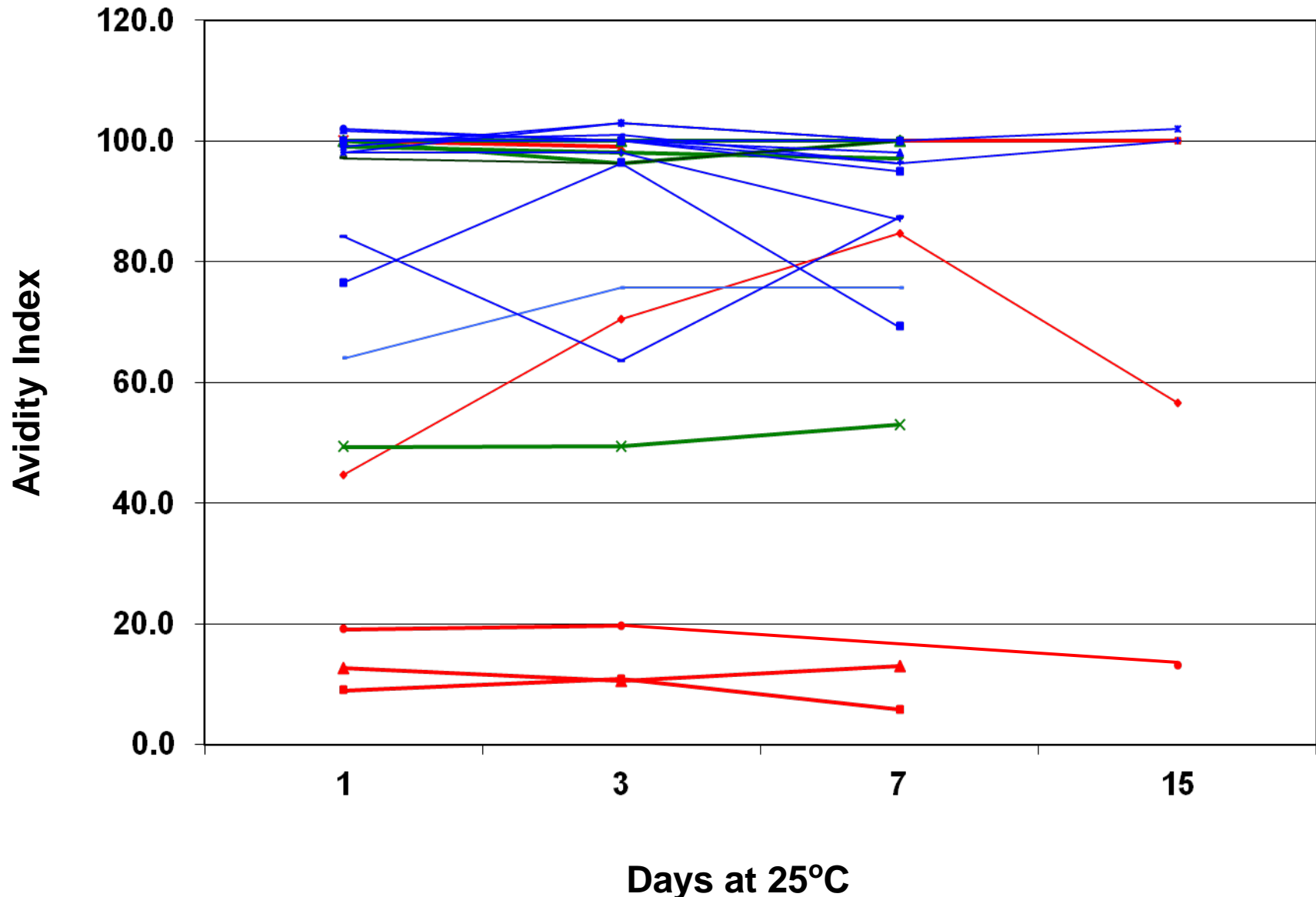

Supplement: Figure S8 — Effect of Days at 25°C on Avidity Assay Results. Samples from subtype C infected individuals from South Africa are marked in green. Samples from subtype A infected individuals from Uganda are marked in orange. Samples from subtype D infected individuals from Uganda are marked in red. Samples from subtype B infected individuals are marked in blue. The x-axis denotes the number of days the sample was incubated at 25°C prior to testing. The y-axis is the assay results as an avidity index. (PDF) [file pone.0025899.s008.pdf]

**Supplementary Figure S9.**

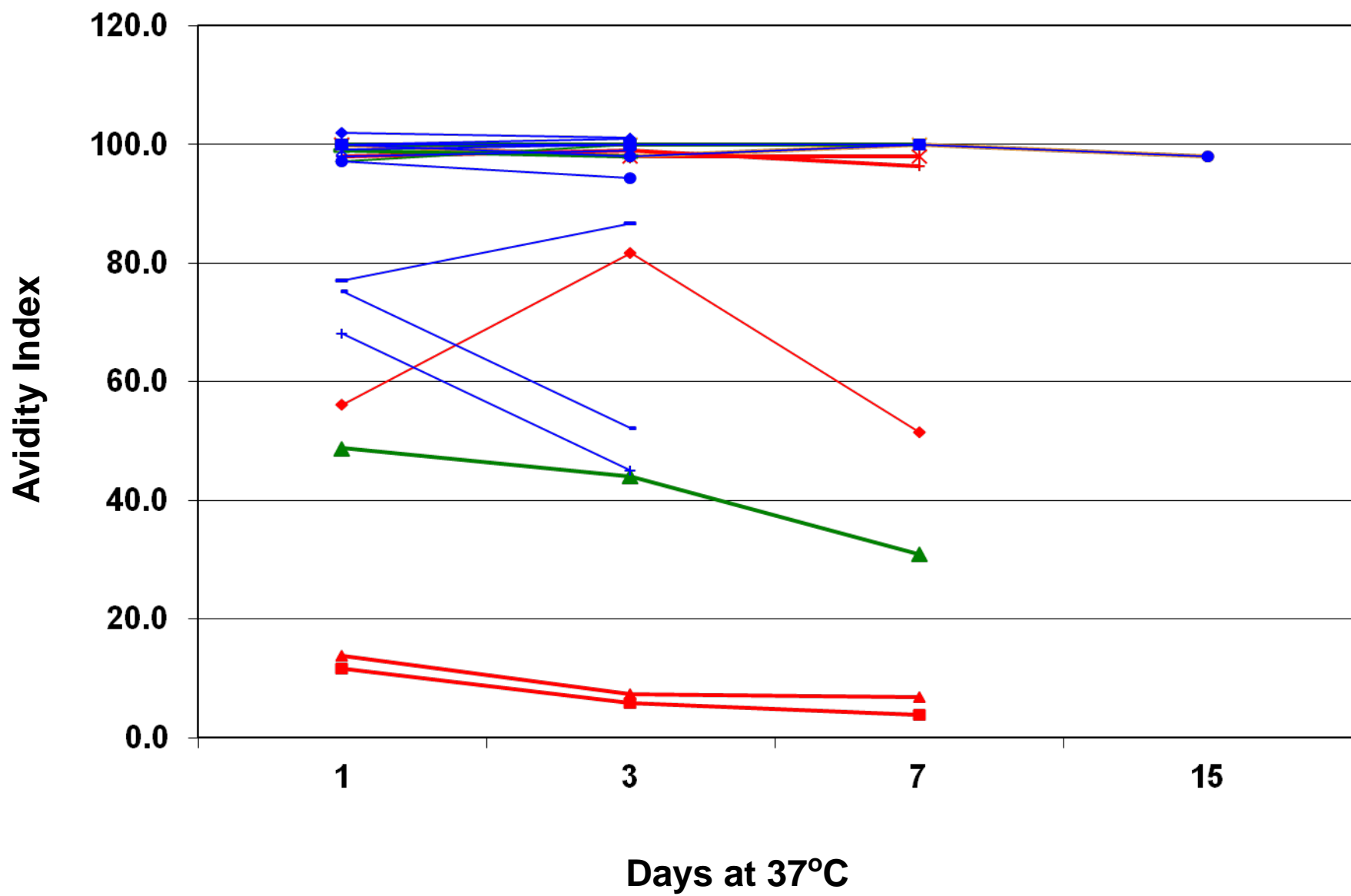

Supplement: Figure S9 — Effect of Days at 37°C on Avidity Assay Results. Samples from subtype C infected individuals from South Africa are marked in green. Samples from subtype A infected individuals from Uganda are marked in orange. Samples from subtype D infected individuals from Uganda are marked in red. Samples from subtype B infected individuals are marked in blue. The x-axis denotes the number of days the sample was incubated at 37°C prior to testing. The y-axis is the assay results as an avidity index. (PDF) [file pone.0025899.s009.pdf]

**Supplementary Figure S10.**

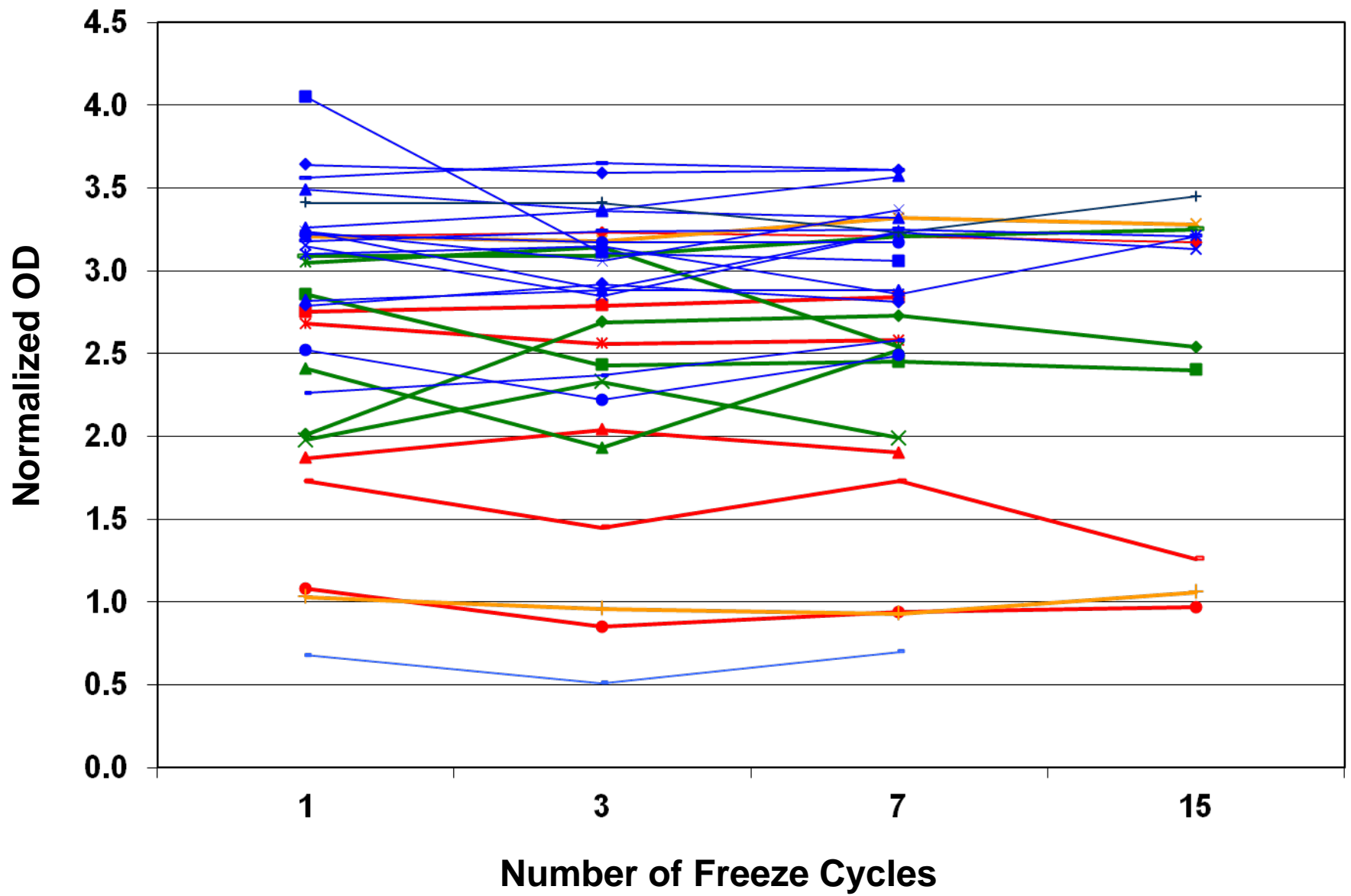

Supplement: Figure S10 — Freeze Thaw Cycles on BED-CEIA Assay Results. Samples from subtype C infected individuals from South Africa are marked in green. Samples from subtype A infected individuals from Uganda are marked in orange. Samples from subtype D infected individuals from Uganda are marked in red. Samples from subtype B infected individuals are marked in blue. The x-axis denotes the number of days the sample was freeze thawed prior to testing. The y-axis is the assay results in normalized optical density units. (PDF) [file pone.0025899.s010.pdf]

**Supplementary Figure S11.**

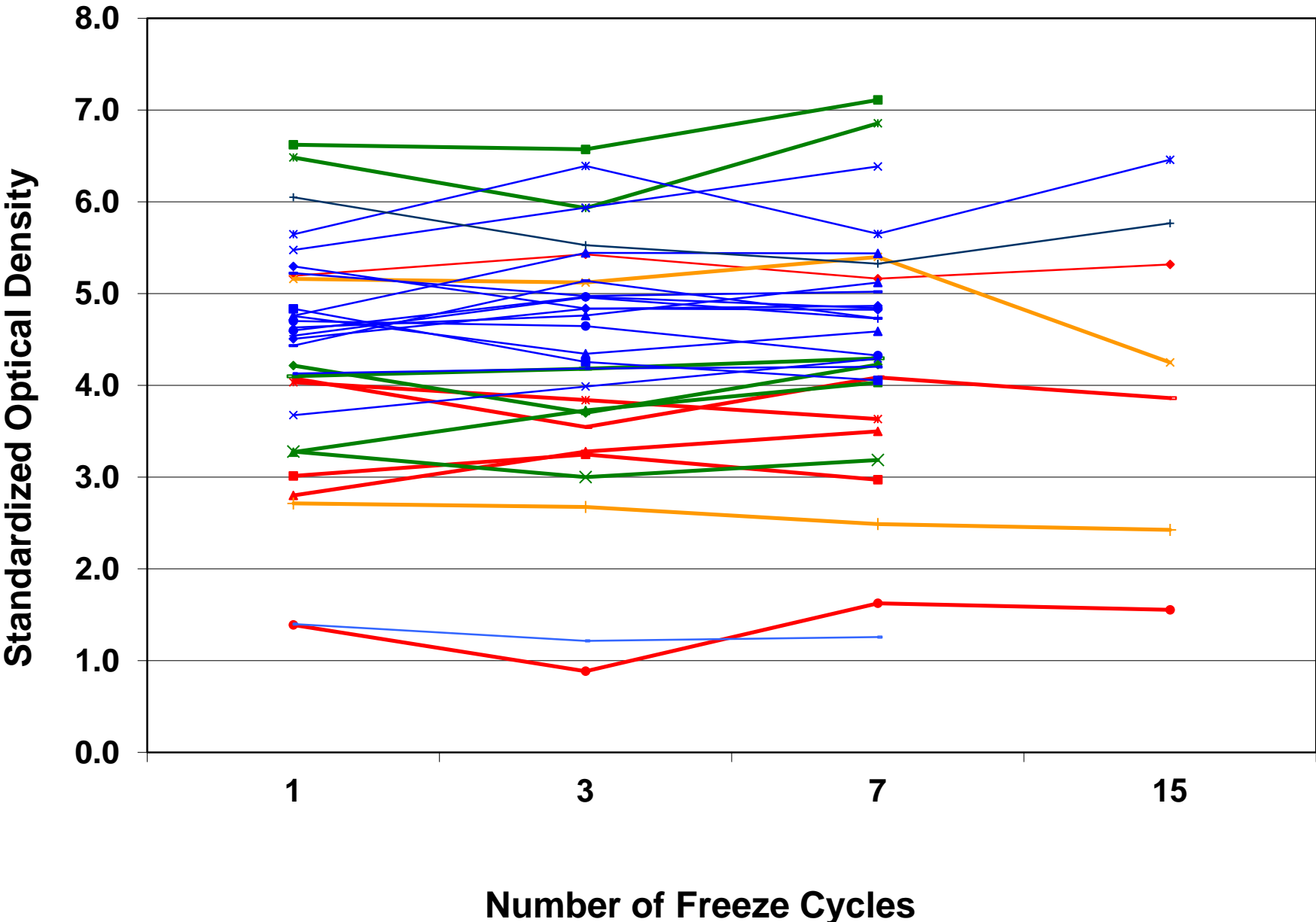

Supplement: Figure S11 — Freeze Thaw Cycles on Vironostika-LS Assay Results. Samples from subtype C infected individuals from South Africa are marked in green. Samples from subtype A infected individuals from Uganda are marked in orange. Samples from subtype D infected individuals from Uganda are marked in red. Samples from subtype B infected individuals are marked in blue. The x-axis denotes the number times the sample was freeze thawed prior to testing. The y-axis is the assay results in standardized optical density units. (PDF) [file pone.0025899.s011.pdf]

Supplementary Figure S12.

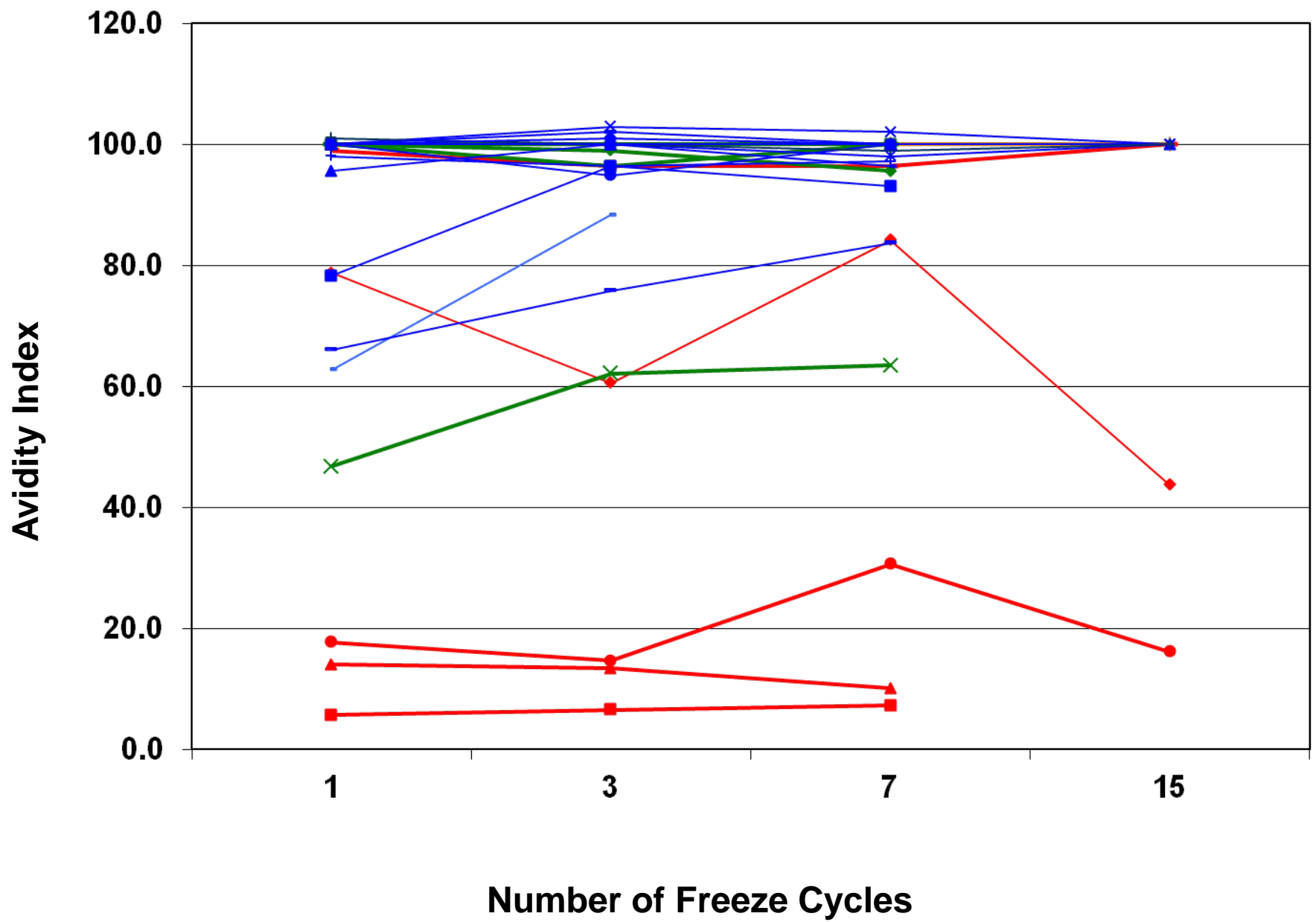

Supplement: Figure S12 — Effect of Freeze Thaw Cycles on Avidity Assay Results. Samples from subtype C infected individuals from South Africa are marked in green. Samples from subtype A infected individuals from Uganda are marked in orange. Samples from subtype D infected individuals from Uganda are marked in red. Samples from subtype B infected individuals are marked in blue. The x-axis denotes the number of days the sample was freeze thawed prior to testing prior to testing. The y-axis is the assay results as an avidity index. (PDF) [file pone.0025899.s012.pdf]
